# HIV-1 Tat induced microglial EVs leads to neuronal synaptodendritic injury: microglia-neuron cross-talk in NeuroHIV

**DOI:** 10.20517/evcna.2022.14

**Published:** 2022-05-31

**Authors:** Muthukumar Kannan, Seema Singh, Divya T. Chemparathy, Abiola A. Oladapo, Dinesh Y. Gawande, Shashank M. Dravid, Shilpa Buch, Susmita Sil

**Affiliations:** ^1^Department of Pharmacology and Experimental Neuroscience, University of Nebraska Medical Center, Omaha, NE 68198, USA.; ^2^Department of Pharmacology and Neuroscience, Creighton University, Omaha, NE 68178, USA.

**Keywords:** NLRP3, microglia-derived EVs, synaptodendritic injury, mEPSC

## Abstract

**Aim:**

Activation of microglial NLRP3 inflammasome is an essential contributor to neuroinflammation underlying HIV-associated neurological disorders (HAND). Under pathological conditions, microglia-derived-EVs (MDEVs) can affect neuronal functions by delivering neurotoxic mediators to recipient cells. However, the role of microglial NLRP3 in mediating neuronal synaptodendritic injury has remained unexplored to date. In the present study, we sought to assess the regulatory role of HIV-1 Tat induced microglial NLRP3 in neuronal synaptodendritic injury. We hypothesized that HIV-1 Tat mediated microglia EVs carrying significant levels of NLRP3 contribute to the synaptodendritic injury, thereby affecting the maturation of neurons.

**Methods:**

To understand the cross-talk between microglia and neuron, we isolated EVs from BV2 and human primary microglia (HPM) cells with or without NLRP3 depletion using siNLRP3 RNA. EVs were isolated by differential centrifugation, characterized by ZetaView nanoparticle tracking analysis, electron microscopy, and western blot analysis for exosome markers. Purified EVs were exposed to primary rat neurons isolated from E18 rats. Along with green fluorescent protein (GFP) plasmid transfection, immunocytochemistry was performed to visualize neuronal synaptodendritic injury. Western blotting was employed to measure siRNA transfection efficiency and the extent of neuronal synaptodegeneration. Images were captured in confocal microscopy, and subsequently, Sholl analysis was performed for analyzing dendritic spines using neuronal reconstruction software Neurolucida 360. Electrophysiology was performed on hippocampal neurons for functional assessment.

**Results:**

Our findings demonstrated that HIV-1 Tat induced expression of microglial NLRP3 and IL1β, and further that these were packaged in microglial exosomes (MDEV) and were also taken up by the neurons. Exposure of rat primary neurons to microglial Tat-MDEVs resulted in downregulation of synaptic proteins- PSD95, synaptophysin, excitatory vGLUT1, as well as upregulation of inhibitory proteins- Gephyrin, GAD65, thereby implicating impaired neuronal transmissibility. Our findings also showed that Tat-MDEVs not only caused loss of dendritic spines but also affected numbers of spine sub-types- mushroom and stubby. Synaptodendritic injury further affected functional impairment as evidenced by the decrease in miniature excitatory postsynaptic currents (mEPSCs). To assess the regulatory role of NLRP3 in this process, neurons were also exposed to Tat-MDEVs from NLRP3 silenced microglia. Tat-MDEVs from NLRP3 silenced microglia exerted a protective role on neuronal synaptic proteins, spine density as well as mEPSCs.

**Conclusion:**

In summary, our study underscores the role of microglial NLRP3 as an important contributor to Tat-MDEV mediated synaptodendritic injury. While the role of NLRP3 in inflammation is well-described, its role in EV-mediated neuronal damage is an interesting finding, implicating it as a target for therapeutics in HAND.

## INTRODUCTION

Globally, 37.7 million people are living with human immunodeficiency virus (HIV) as of 2020, with greater than 50% having access to combined antiretroviral therapy (cART) as of June 2021 (UNAIDS). The introduction of cART has changed the face of HIV-1 from a death sentence to a manageable chronic disease condition. HIV enters the brain soon after infection via Trojan horse mechanisms involving the migration of infected monocytes across the blood-brain barrier (BBB)^[1]^. It has been demonstrated that HIV patients on cART with no detectable viral load therapy^[2]^ continue to be inflicted with complications of the central nervous system (CNS), collectively termed HIV-associated neurocognitive disorders (HAND). This is likely due to the presence of ongoing low-level virus replication and HIV-1 viral proteins in the brain. About half of the HIV-1 infected patients go on to develop HAND regardless of the cART regimen^[3]^, thereby implicating the effects of residual viral proteins on the CNS^[4]^. Among these viral proteins, the trans-activator of transcription (Tat) is an early viral regulatory protein that enhances the efficiency of viral transcription in the brain^[5]^. Tat is an HIV-1 protein with a variable length of 86-102 amino acids^[6,7]^ and plays a critical role in HIV pathogenesis owing to its cytotoxic potential^[8]^.

In the CNS, HIV does not directly infect the neurons but can efficiently infect microglia^[9]^ and, to some extent, the astrocytes^[10,11]^. HIV-infected microglia or astrocytes, in turn, produce Tat protein, which then can be taken up by the uninfected bystander cells, including the neurons^[12-14]^. A sufficient amount of Tat is present in the CNS of HIV-infected patients to induce neurotoxicity and neuronal dysfunction *in vivo*^[15]^ as well as *in vitro *in cultured neurons^[16]^. Reports have shown that Tat alters the expression of neuronal proteins such as the postsynaptic density protein 95 (PSD-95)^[17]^, Gephyrin^[17]^, and synaptophysin^[18]^.

Microglia not only function as the resident immune cells of the CNS, but also communicate with various other CNS cells, including the neurons^[19]^ and astrocytes^[20]^, for normal functioning of the brain. In healthy cells, microglia secrete extracellular vesicles (EVs) to support the metabolic functions of neurons and to provide substrates needed for energy metabolism during synaptic activity^[21-25]^. Similarly, under pathological conditions, microglia-derived EVs (MDEVs) can also affect neuronal functions by delivering proinflammatory cytokines and other neurotoxic mediators to these latter cells^[26,27]^. Previous reports from our laboratory have shown that HIV-1 Tat could impact the astrocyte EV cargo, which in turn, could impair the synaptic architecture of neurons^[28,29]^. It has also been suggested that secretion of EVs could be a necessary and compensatory pathway to eliminate damaged or toxic molecules produced due to Tat cytotoxicity^[30-33]^. To support this notion, we and others have shown that HIV-1 Tat inhibits the autophagy and proteasomal degradation pathways in microglia and astrocytes^[30-33]^, which in turn, can modulate the EV biogenesis pathways^[34,35]^. Additionally, our previous study has demonstrated that HIV-1 Tat can induce the NLRP3 inflammasome pathway in microglia^[36]^, resulting in their activation. Taken together, we thus hypothesized that in the context of HIV/HAND pathology EVs released from inflammasome-activated microglia cells could also carry the inflammatory mediators such as NLRP3, which, upon being taken up by the neurons, could affect their functions.

In the present study, we isolated and characterized EVs released by BV2 cells (immortalized murine microglial cells) and human primary microglia (HPM) cells in the presence or absence of HIV-1 Tat. Both the BV2 and primary microglial cells derived-MDEVs were demonstrated to carry the NLRP3 and IL1β cargoes that, upon being uptaken by the neurons, resulted in synaptodendritic injury and lowering excitatory postsynaptic currents, suggesting that the MDEVs via an NLRP3 dependent mechanism could be a contributing factor for HAND pathogenesis.

## METHODS

### Reagents

NLRP3 (AG-20B-0014, AdipoGen, CA, USA); IL1β (ab9722, Abcam, MA, USA); Anti-CD63 antibody (ab216130, Abcam, MA, USA); Anti-CD9 antibody (ab92726, Abcam, MA, USA), Anti-TSG101 antibody (ab125011, Abcam, MA, USA), Anti-Alix antibody (ab275377, Abcam, MA, USA), Anti-Calnexin antibody (ab133615, Abcam, MA, USA); Anti-PSD95 antibody (ab2723, Abcam, MA, USA); Anti-GAD65 antibody (ab239372, Abcam, MA, USA); Anti-Gephyrin antibody (ab181382, Abcam, MA, USA); Anti-vGLUT1 antibody (AB5905, Millipore, Burlington, MA, USA), GFP expressing plasmid (13031, Adgene, Watertown, MA, USA); β-actin (A5316, Sigma- Aldrich, MO, USA); horseradish peroxidase conjugated goat anti-rabbit (sc-2004, Santa Cruz Biotechnology, TX, USA) and horseradish peroxidase conjugated goat anti-mouse (sc-2005, Santa Cruz Biotechnology, TX, USA); human primary microglia (Cat # 1900; Sciencell research laboratory, CA, USA) and BV2 microglial cell line was received from Dr. Sanjay Maggirwar (University of Rochester Medical Center, Rochester, NY, USA).

### Microglia culture 

HPM cells were purchased from Celprogen (cat no:37089-01). HPM cells were grown in HPM culture complete Media with Serum (Cat. No: M37089-01, Celprogen, CA, USA). The BV2 cell lines were obtained from Dr. Sanjay Maggirwar (University of Rochester Medical Center, Rochester, NY, USA). These cultured BV2 cells were maintained in Dulbecco’s modified Eagle’s medium (DMEM) with high glucose supplemented with 10% heated-inactivated fetal bovine serum, penicillin (100 units/mL), and streptomycin (100 μg/mL). Both the cell types were seeded at a density of 3 × 10^5 ^(per well) in a six-well plate or 2.5 × 10^6^ in a T150 flask for different experiments. After overnight serum starvation, cells were treated with 50 ng/mL HIV-1 Tat for 24 h in a humidified incubator at 37 °C with 5% CO_2_, followed by EV isolation.

### Rat primary neuron cultures

Rat primary cortical and hippocampal neurons were isolated from E18 rats as described previously^[37,38]^. The animal experiment is approved by the ethical committee of University of Nebraska Medical Center (IACUC# 20-057-07-FC). Briefly, the cortex and hippocampi were dissected in HBSS (14025076, Gibco™) followed by incubation with 0.25% trypsin (25200056, Gibco™) for 10 min in a 37 °C water bath. Tissue-free, single cell suspensions were obtained by triturating, followed by passing the cell suspension through the 40 µM cell strainer. Primary neuron cultures were maintained in a complete neuronal media containing neuro­basal medium (21103049, Invitrogen), with B27 supplement (17504044, Invitrogen), L-Glutamate (A2916801, Gibco™), and penicillin-streptomycin (15070063, Invitrogen). Rat primary cortical neurons were seeded at a density of 2 × 10^5 ^in poly-D-lysine (P1024, Sigma-Aldrich) coated plates for western blot analysis. For microscopy analysis, hippocampal neurons were seeded at a density of 1.5 × 10^5^ in poly-D-lysine coated coverslips. After two weeks of culture, cells were used for further experimentation.

### EV isolation

The EVs were prepared from the supernatant of BV2 cells and HPM by differential centrifugations, which was previously described^[29]^. Briefly, serum-starved BV2 cells and HPM were exposed to HIV-1 Tat protein (50 ng/mL) for 24 h. Then, the conditioned media from this treatment were harvested, centrifuged at 300 *g* and 2000 *g* for 10 min to eliminate cellular debris and residual cells, and the supernatant was spun at 10,000 *g* for 30 min, followed by filtration using a 0.22 μm filter. The EVs were pelleted by ultracentrifugation (Beckman Ti32 rotor, Brea, CA, USA) for 70 min at 100,000 *g*. All EV isolation protocols were performed at 4 °C. The EVs were quantified using the ZetaView nanoparticle tracking analysis system (NS300, Particle Metrix, Germany) as described previously^[29]^. The protein content was assessed using a BCA protein assay kit (Pierce, Rockford, IL, USA); after normalization, the EVs were used for characterization of the exosome-specific markers by Western blotting and transmission electron microscopy, as well as used for further experimentation. The total number of EVs from 2 million cells were diluted in 300 µL of PBS. Approximately 2 million BV2/HPM cells yielded 10^9^/mL of EVs. All the neurons were exposed with 100, 500, and 1000 EVs/ cell for standardization, and 500 EVs/ neurons were used for further experimentation (after standardization). The neurons were exposed to these EVs for 48 h, followed by an assessment of synaptodendritic injury and electrophysiology.

### Zeta view tracking analysis 

Isolated EVs from BV2 or HPM supernatant were analyzed by nanoparticle tracking analysis (NTA) using ZetaView nanoparticle tracking analyzer (Particle Metrix, Germany) along with the software ZetaView 8.04.02 SP1. Prior to the analysis, the instrument was calibrated using 100 nm polystyrene nano standard particles and cell quality checking was performed before sample reading. The video was captured at a sensitivity of 85, a shutter speed of 100, and a frame rate of 30. Size (in nm) and concentration (particles/mL) for each sample were determined by injecting the diluted sample in filtered PBS, with two cycles of reading at each position.

### Electron microscopy

EV pellets were subjected to negative staining. In brief, EV pellets were deposited on 200-mesh Formvar-coated copper grids and the membranes were covered for 4-5 min for the absorption. Next, for contrast staining, the grids were further transferred to uranyl acetate solution. Hereafter, the grids were washed with PBS and excess fluid was blotted with filter paper and allowed to air-dry at room temperature. Imaging was performed using a Hitachi H-7500 electron microscope (Hitachi, Tokyo, Japan) at 200kV.

### NLRP3 siRNA transfection

Microglial cells were seeded at a density of 3 × 10^5 ^(per well) in a 6-well plate for siRNA transfection. At about 70% confluency, cells were transfected with NLRP3 siRNA or scrambled siRNA in Opti-MEM media (Life Technologies, 31985062) using Lipofectamine RNAiMAX transfection reagent (Thermo Fisher Scientific, catalog: 13778150) according to the manufacturer’s instructions. Following transfection, cells were incubated for 12-18 h in a humidified incubator at 37 °C with 5% CO_2_. After transfection, cells were treated with 50 ng/mL Tat in a fresh DMEM medium for 24 h. The siRNA transfection efficiency was determined by western blotting. 

### GFP-plasmid transfection

Lipofectamine 3000 (2307436, Invitrogen, Carlsbad CA) was used for GFP plasmid transfection in hippocampal (0.15 × 10^6 ^cells/well) neurons according to the manufacturer’s instructions. Briefly, cells were transfected with GFP plasmid (500 ng) mixed with 1μl of Lipofectamine 3000 diluted in 25 μL of Opti-MEM (31985-070, Gibco, Thermo Fisher Scientific, Amarillo, TX) media. The cells were incubated with the plasmid-lipid complex for 6 h, and the medium was changed with fresh media. Thereafter, transfected cells were used for various treatments after 72 h.

### Immunocytochemistry

GFP-plasmid transfected primary rat hippocampal neurons on coverslips were exposed to MDEVs for 48 h. After the treatment, neurons were fixed with 4% paraformaldehyde (PFA) for 20 min at room temperature and permeabilized with 0.1% Triton X-100 (BP151-1; Thermo Fisher Scientific, Grand Island, NY, USA). Blocking was performed with 5% normal goat serum in PBS for 1 h at room temperature, followed by the addition of respective primary antibodies: GAD65 (ab18258, Cambridge, MA), vGlut1 (ab5905, Cambridge, MA). After incubation with primary antibody overnight, the neurons were probed with secondary Alexa Fluor 594 goat anti-rabbit (A11012, Invitrogen, Carlsbad CA) and Alexa Fluor 647 goat anti-guinea pig (A21450, Invitrogen, Carlsbad CA) antibodies. Cells were counterstained and mounted with Prolong Gold antifade reagent with DAPI (P36935, Invitrogen, Carlsbad CA).

### Image acquisition and dendritic spine quantification

Images were captured in confocal microscopy (ZEISS ELYRA PS.1 Super Resolution Microscope, Jena, Germany) with a 63× magnification with consistent contrast and brightness for each set of experiments. Dendritic spines were captured using Z-stack projection. Thereafter, Sholl analysis was performed for analyzing dendritic spines using neuronal reconstruction software Neurolucida 360 (version 2021.1.1).

### Western blotting

The BV2 or HPM cells treated with HIV-1 Tat in culture were lysed using the mammalian cell lysis kit (Sigma, MCL1-1KT). Protein electrophoresis was performed using 10 μg of the lysate proteins on a 10% sodium dodecyl sulfate-polyacrylamide gel under reducing conditions, then transferred to polyvinylidene difluoride (PVDF) membranes (Millipore, IPVH00010). Then, the blots were blocked with 5% nonfat dry milk diluted in 1 × TTBS buffer. After washing with 1 × TTBS buffer three times, the membranes were probed with primary antibodies specific for the proteins of interest overnight. The secondary antibodies used to probe were HRP conjugated to goat anti-mouse/rabbit IgG. β-actin (Sigma, A5441) was used as a loading control for the study.

### Electrophysiology

Whole-cell electrophysiology was performed on rat primary hippocampal neurons (DIV 19-21) as previously described^[39]^. Primary hippocampal neurons were seeded on coverslips. The signal was filtered at 2 kHz & digitized at 10 kHz using an Axon Digidata 1440A analog-to-digital board (Molecular Devices, San Jose, CA, USA). Recordings with a pipette access resistance of less than 20 mOhm and less than 20% changes during the duration of recording were included. The external solution contained (in mM): 150 NaCl, 3 KCl, 10 HEPES, 6 mannitol, 0.02 EDTA, 1.5 MgCl_2_, and 2.5 mM CaCl_2_ (pH 7.4). Glass pipettes with a resistance of 2-5 mOhm were filled with an internal solution consisting of (in mM) 110 cesium gluconate, 30 CsCl, 5 HEPES, 4 NaCl, 0.5 CaCl_2_, 2 MgCl_2_, 5 BAPTA, 2 Na_2_ATP, and 0.3 Na_2_GTP (pH 7.35). QX-314 was added in pipette solution to block voltage-gated sodium channels. Miniature excitatory postsynaptic currents (mEPSCs) were recorded in the presence of 0.5 μM tetrodotoxin and 100 μM picrotoxin at -70 mV. The mEPSC recordings were analyzed using Minianalysis software (Synaposoft, Atlanta, GA, USA) with an amplitude threshold set at 5 pA. The frequency of the miniature currents was measured.

### Statistics

The data are represented as mean ± SEM. Student t-test was employed to compare between two groups, and one-way ANOVA followed by Bonferroni post hoc test was employed to compare within multiple experimental groups, using the GraphPad Prism software (Version 5). For the *in vitro* study, three replicates per sample and six independent sets of experiments were analyzed. Statistical analysis for which probability levels were less than *P *< 0.05 was considered statistically significant.

## RESULTS

### HIV-1 Tat increases NLRP3 cargoes in exosomes derived from microglia 

**Figure 1:**
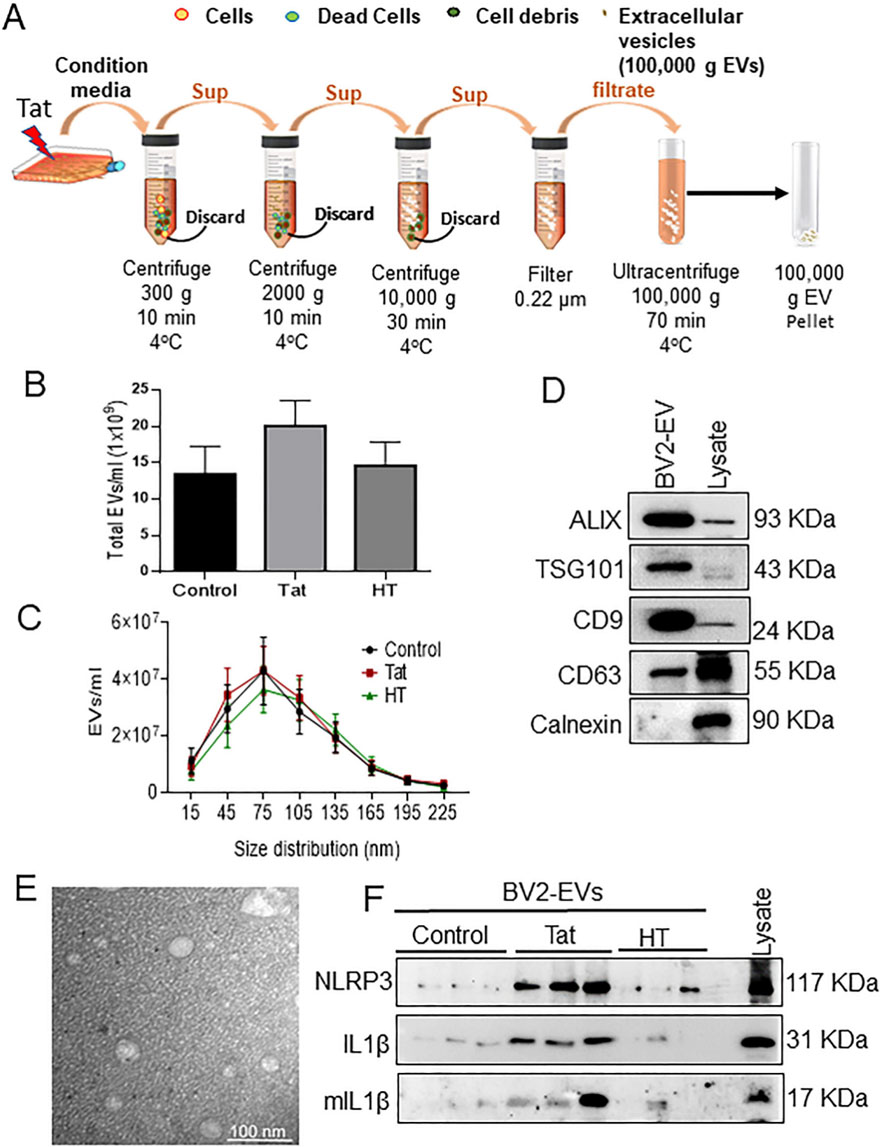
Characterization of microglia-derived exosomes from BV2 cells. (A) Schematic representation of the isolation protocol from BV2 cells. (B) Quantification of the total number of EVs and (C) size distribution of exosomes by NTA using ZetaView. (D) Representative western blots showing the expression of exosome-specific markers (Alix, TSG101, CD9, CD63). Calnexin used as a negative control for exosomes. (E) Representative transmission electron microscopy (TEM) image of exosome particles isolated by ultracentrifugation at 100,000 g. Scale bar 100 nm. (F) Representative western blot images showing protein expression of NLRP3, pro-IL1β, mIL-β in control, Tat (50 ng/mL) or HT treated-BV2-derived exosomes. Data are presented as mean ± SEM. NLRP3: NOD-, LRR- and pyrin domain-containing protein 3; IL: interleukin; mIL: mature interleukin; Tat: trans-activator of transcription; HT: heat inactivated Tat protein.

**Figure 2:**
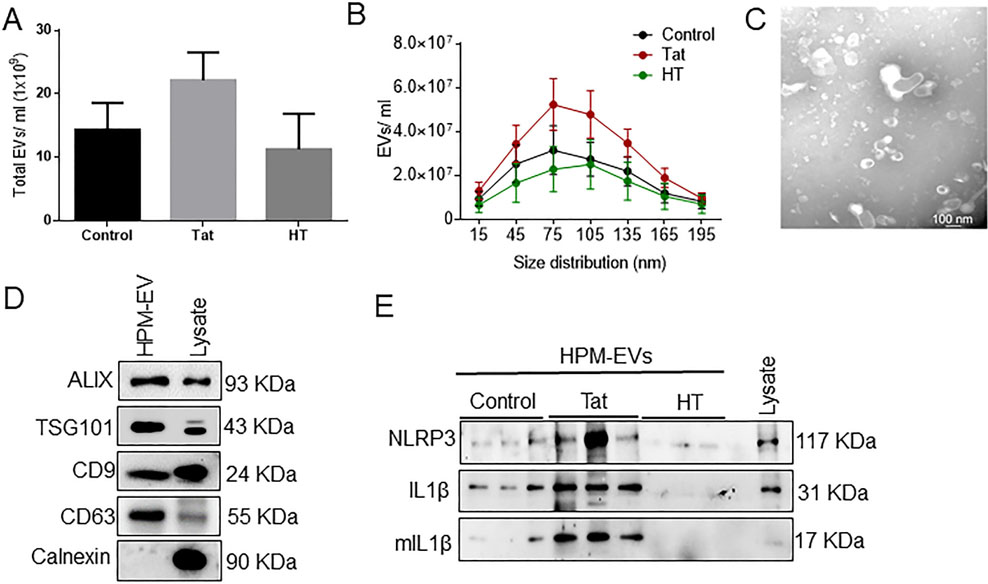
Characterization of microglia-derived exosomes from human primary microglia (HPM). (A) Quantification of the total number of EVs and (B) size distribution of exosomes by NTA using ZetaView. (C) Representative TEM image of exosome particles isolated by ultracentrifugation at 100,000 g. Scale bar 100 nm. (D) Representative western blots showing expression of exosome-specific markers (Alix, TSG101, CD9, CD63). Calnexin is used as a negative control for exosomes. (E) Representative western blot images showing protein expression of NLRP3, pro-IL1β, mIL-1β in control, Tat (50 ng/mL) or HT treated-HPM-derived exosomes. Data are presented as mean ± SEM. Abbreviations: similar as Figure 1.

In our previous study, we demonstrated the role of HIV-1 Tat in activating NLRP3 inflammasome with subsequent maturation of caspase-1 and production and release of IL-1β from microglia^[36]^. NLRP3 inflammasome plays a crucial role in the development of neuroinflammation^[40]^. It has been shown that microglial exosomes can be transported and taken up by the recipient cells^[24,25,41]^; however, scanty reports are available on the functionality of these microglial exosomes, specifically in the context of HIV-1. In the current study, we investigated whether microglial NLRP3 plays a role in causing neuronal damage via microglia-neuron cross-talk involving the exosomes. We first sought to isolate and characterize EVs from conditioned media of BV2 cells and HPM with or without Tat exposure [Figure 1A]. The total number of EVs and particle size distribution were determined by NTA. Exposure of BV2 cells [Figure 1B] and HPM [Figure 2A] to HIV-1 Tat (50 ng/mL, 24h) resulted in increased release of exosomes compared to the control or heated Tat (HT) exposed cells; however, there was no significant difference among the groups. Size distribution by NTA showed that isolated EVs were in the size range of 50-150 nm in BV2 cells [Figure 1C] and HPM [Figure 2B]. The protein expression of exosomal markers such as Alix, TSG101, CD9, and CD63 was analyzed by western blotting in BV2 [Figure 1D] and HPM [Figure 2D] EVs. Additionally, immunoblotting of calnexin was also performed to demonstrate that isolated EVs were pure and enriched vesicles [Figures 1D and 2D]. Further characterization by TEM revealed the cup-shaped profile of EVs with sizes ranging from 50-150 nm in BV2 [Figure 1E] and HPM [Figure 2C]. Since we were specifically interested in the role of NLRP3 inflammasome pathway, we determined the protein expression of NLRP3 and its downstream IL1β in the exosomes derived from BV2 and HPM. HIV-1 Tat was found to increase the release of NLRP3, pro- and mature IL1β in EVs isolated from BV2 cells [Figure 1F] and HPM [Figure 2E].

### BV-2 exosomes cause neuronal damage 

**Figure 3:**
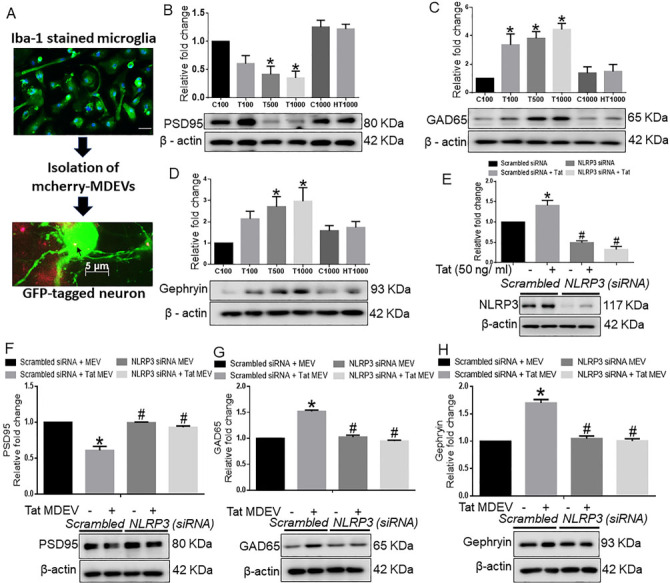
Role of BV2 derived exosomes from BV2 on neuronal synaptic proteins. (A) Schematic representation showing the exposure of BV2-derived exosomes to primary neurons. Representative western blot images and their quantitative analysis showing the dose-dependent effects of BV2-derived exosomes on the expression of (B) PSD95, (C) GAD65, and (D) Gephyrin in rat cortical primary neurons. (E) Representative western blot images and quantitative analysis showing the expression of NLRP3 in BV2 cells transfected with either NLRP3 or scrambled siRNA in the presence of Tat (50 ng/mL) to confirm the transfection efficiency of NLRP3. Representative western blots and their quantitative analysis showing the expression of (F) PSD95, (G) GAD65, and (H) Gephyrin in rat primary cortical neurons exposed with MDEVs from NLRP3 siRNA transfected BV2 cells in the presence of Tat (50 ng/mL). β-actin was used as a loading control. Data are presented as mean ± SEM. *P < 0.05 *vs.* siControl MDEV, ^#^*P* < 0.05 *vs*. Tat MDEV. One-way ANOVA followed the Bonferroni post hoc tests were used for statistical analysis. MDEV: Microglia derived exosomes’ HT: heated Tat; NLRP3: NOD-, LRR- and pyrin domain-containing protein 3; PSD95: postsynaptic density protein 95; GAD65: glutamic acid decarboxylase; siRNA: small interfering RNA; Tat: trans-activator of transcription.

Since excessive microglial activation damages the surrounding healthy cells, we next enquired whether factors derived from activated microglia could reach the recipient neurons and inflict neuronal damage. For this, BV2 cells were transfected with a plasmid encoding the exosome marker TSG101 fused with mCherry, followed by isolation of EVs from the conditioned media of transfected BV2 cells. As shown in Figure 3A, BV2-derived exosomes were found localized within the neurons. Having confirmed the transfer of exosomes from microglia to neurons, we next asked whether NLRP3 carried through the exosomes could be taken up by the neurons and inflict neuronal damage. For this, rat cortical neurons were exposed to exosomes isolated from conditioned media of BV2 cells with or without Tat exposure. As neuronal excitability relies precisely on excitatory and inhibitory signals, we analyzed the protein expression of postsynaptic density protein 95 (PSD95), a critical synaptic protein that controls synaptic transmission and plasticity. Figure 3B represents the immunoblotting analysis of PSD95, which showed a dose-dependent significant down-regulation of PSD95 in cortical neurons exposed to exosomes isolated from Tat treated BV2 cells (*P *< 0.05) compared with control EV exposed neurons. Interestingly, there was a significant increase in the expression of inhibitory postsynaptic markers, glutamic acid decarboxylase 65 (GAD65), in the neurons exposed to varying numbers of Tat-MDEVs (*P *< 0.05) compared with neurons exposed to control MDEVs [Figure 3C]. Similarly, the expression of inhibitory postsynaptic markers, Gephyrin, was also found to be significantly upregulated in neurons exposed to Tat-MDEVs (*P *< 0.05) compared with control MDEVs [Figure 3D].

While the neurotoxic effect of NLRP3 has been well documented in neurons^[41]^, the direct role of microglial NLRP3 on neuronal synaptodendritic injury has not been shown to date. To assess this phenomenon, the expression of NLRP3 was first silenced in BV2 cells using the siRNA approach [Figure 3E]. Next, to determine the transfection efficiency, NLRP3 expression was assessed in different groups. Results showed that NLRP3 expression was significantly increased in the Tat exposed BV2 cells compared with the scrambled control group, and that NLRP3 expression was minimal in the siNLRP3 transfected groups in the presence or absence of Tat (50 ng/mL, 24 h) (*P *< 0.05) [Figure 3E]. As shown in Figure 3F, Tat-MDEVs significantly downregulated the expression of PSD95 compared with the control-MDEV group. Silencing of microglial NLRP3 significantly abrogated Tat-MDEV mediated downregulation of PSD95 in cortical neurons (*P *< 0.05). Similarly, the expression of GAD65 [Figure 3G] and Gephyrin [Figure 3H] was significantly downregulated in neurons exposed to NLRP3 silenced MDEVs exosomes compared with neurons exposed to Tat-MDEVs (*P* < 0.05).

### Microglial NLRP3 leads to neuronal dendritic injury 

**Figure 4:**
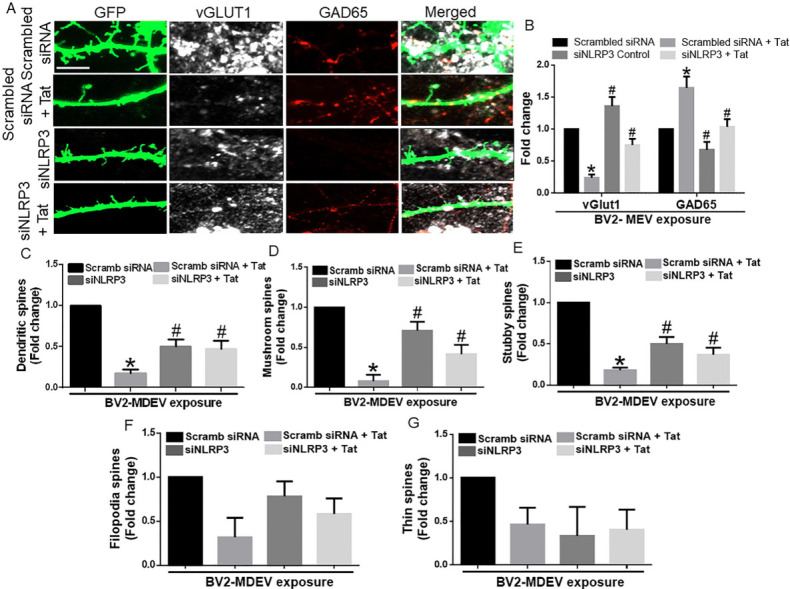
Role of BV2-derived exosomes on neuronal dendritic spines. (A) Representative immunofluorescence images showing hippocampal spine density & expression of vGlut1 and GAD65, after exposure of hippocampal neurons with MDEVs from control, Tat, NLRP3 silenced and NLRP3 silenced- Tat treated BV2 cells. Scale bar: 2 µm. (B) Quantitative analysis of vGlut1 and GAD65 via Image J Launcher software. (C) Quantification of spine numbers in different groups of neurons via Neurolucida software. (D-G) Quantification of spine sub-types in different groups of neurons via Neurolucida software. Data are presented as mean ± SEM. *P < 0.05 *vs.* siControl MDEV, ^#^*P* < 0.05 *vs.* Tat-MDEV. One-way ANOVA, followed by the Bonferroni post hoc tests, was used for statistical analysis. Scramb: scrambled siRNA (small interfering RNA); NLRP3: NOD-, LRR- and pyrin domain-containing protein 3; vGLUT1: vesicular glutamate transporters; GAD65: glutamic acid decarboxylase; Tat: trans-activator of transcription.

Having demonstrated that Tat stimulated BV2-derived MDEVs induced alterations in the expression of synaptic proteins, we next wanted to assess the role of these MDEVs in mediating neuronal dendritic injury. As expected, and as demonstrated in Figure 4A, synaptic spines were abundantly present in the hippocampal neurons exposed to BV2-siControl MDEVs. There was, however, significant downregulation (*P* < 0.05) of neuronal spines in neurons exposed to BV2 Tat-MDEVs. Interestingly, neurons exposed to microglial NLRP3 silenced Tat-MDEVs showed restoration of spine numbers similar to the control group [Figure 4C]. Interestingly, the expression of the vGLUT1 was significantly (*P* < 0.05) decreased and that of the GAD65 increased in the hippocampal neurons exposed to MDEVs derived from scrambled siRNA+Tat treated BV2 cells, while both of these synaptic proteins remained unchanged in MDEV exposed neurons from NLRP3 silenced BV2 [Figure 4B]. Another important finding of this study was that the most mature spine sub-type, the mushroom type, was present in high numbers in neurons exposed to MDEVs isolated from scrambled siRNA treated BV2 cells, while they decreased significantly in the neurons exposed to MDEVs isolated from scrambled siRNA+Tat treated BV2 cells (*P* < 0.05) [Figure 4D]. A similar trend was also observed for the stubby spines [Figure 4E]. MDEVs isolated from NLRP3 silenced BV2 cells, however, showed a similar trend as that of the scrambled siRNA treated BV2 cells for mushroom and stubby spines [Figure 4D-E]. The numbers of immature filopodial and thin spines, however, did not change significantly across the different groups [Figure 4F-G].

### HPM exosomes cause neuronal synaptodendritic injury 

**Figure 5:**
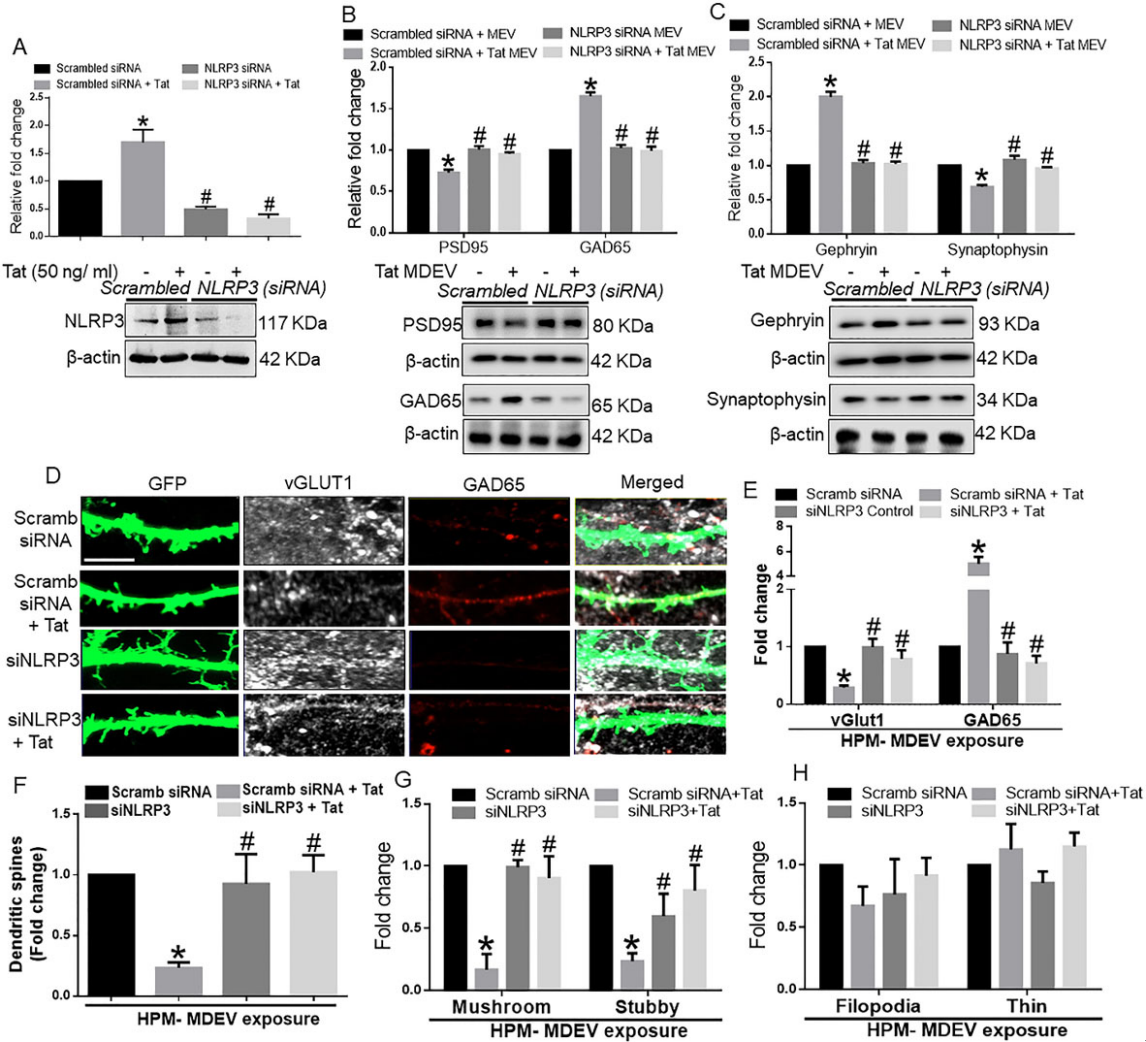
Role of human primary microglia-derived exosomes on neuronal miniature excitatory postsynaptic currents (mEPSC). (A) Representative traces of whole-cell voltage-clamp recording showing mEPSC, (B) mean mEPSC frequencies (Hz), and (C) amplitude (pA) in primary rat hippocampal neurons (DIV 18-22) exposed to MDEVs from control, Tat, NLRP3 silenced and NLRP3 silenced- Tat treated HPM cells. Data are presented as mean ± SEM. *P < 0.05 *vs.* siControl MDEV, ^#^*P* < 0.05 *vs.* Tat MDEV. One-way ANOVA, followed by the Bonferroni post hoc tests, was used for statistical analysis. Scramb: Scrambled siRNA (small interfering RNA); MDEV: microglia derived exosomes; NLRP3: NOD-, LRR- and pyrin domain-containing protein 3; PSD95: postsynaptic density protein 95; vGLUT1: vesicular glutamate transporters; GAD65: glutamic acid decarboxylase; siRNA: small interfering RNA; Tat: trans-activator of transcription.

We next wanted to validate our findings with HPM-derived EVs. We assessed the role of NLRP3 in HPM-derived EVs on a neuronal synaptodendritic injury. Similar to BV2 cells, neurons were also exposed to HPM-derived EVs for 48 h. As shown in Figure 5A, in HPMs transfected with either scrambled or NLRP3 siRNA, Tat significantly (*P* < 0.05) increased NLRP3 expression in HPM. In NLRP3 silenced cells, there was effective NLRP3 silencing. As shown in Figure 5B, HPM-Tat-EVs significantly (*P* < 0.05) downregulated the expression of PSD95 and upregulated the expression of GAD65 in neurons compared with neurons exposed to control MDEVs. In neurons exposed to MDEVs from microglial silenced NLRP3, on the other hand, Tat failed to alter the expression of synaptic proteins (*P* < 0.05). Similarly, in neurons exposed to HPM-Tat-MDEVs, significantly (*P* < 0.05) downregulated the expression of synaptophysin and upregulated the expression of Gephyrin in neurons compared to neurons exposed to control MDEVs. MDEVs from NLRP3 silenced HPM in the presence/absence of Tat showed minimal alterations in the expression of synaptic proteins (*P* < 0.05) [Figure 5C]. Having demonstrated that Tat stimulated HPM-derived EVs induced alterations in the expression of the synaptic proteins, we next wanted to assess the role of these exosomes in neuronal dendritic injury. As demonstrated in Figure 5D, there was an abundant expression of synaptic spines in hippocampal neurons exposed to MDEVs isolated from HPMs treated with scrambled siRNA. Total spine numbers, however, were significantly downregulated (*P* < 0.05) in neurons exposed to MDEVs isolated from HPM transfected with scrambled siRNA+Tat group. Neurons exposed to MDEVs from NLRP3 silenced group showed spine numbers similar to the control group [Figure 5F]. Interestingly, the expression of the vGLUT1 was significantly (*P* < 0.05) decreased, and that of the GAD65 increased in hippocampal neurons exposed to MDEVs isolated from HPM transfected with scrambled siRNA+Tat group. In neurons exposed to MDEVs from NLRP3 silenced HPMs, the expression levels of both the synaptic proteins were comparable to the neurons exposed to control MDEVs [Figure 5E]. Intriguingly, we found that the numbers of mushroom and stubby spines were significantly (*P* < 0.05) decreased (*P* < 0.05) in the neurons exposed to MDEVs isolated from the HPM+Tat group compared to that of control [Figures 5G]. As expected, the NLRP3 silenced groups showed a similar trend as that of the control group [Figure 5G]. However, the immature thin and filopodial spines did not significantly change across the different groups [Figure 5H].

### HPM exosomes reduce excitatory neurotransmission 

**Figure 6:**
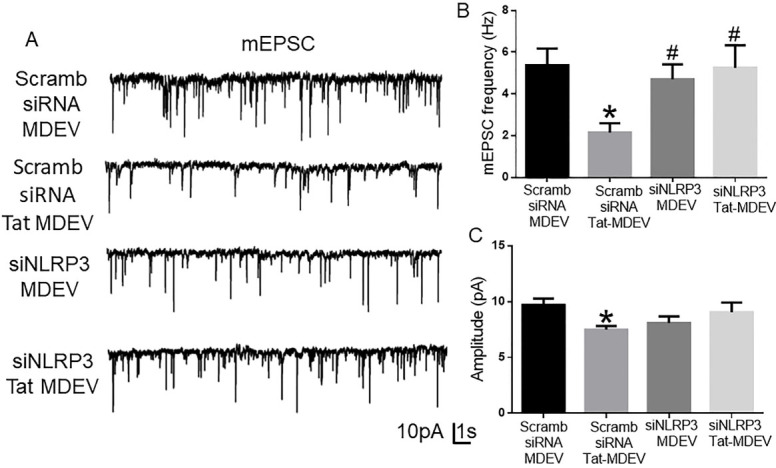
Schematic representation of the role of microglial NLRP3 on neuronal synaptodendritic injury via exosomes. Exposure of microglia (BV2 or HPM) with HIV-1 Tat results in the activation of NLRP3 inflammasome complex, whichleads to the production of IL-1β and microglial activation. Thereafter, these NLRP3 and IL1β can be packaged in the exosomes and released by the microglia. These exosomes carrying NLRP3/IL1β upon uptake by the neurons result in alteration of synaptic proteins (PSD95, vGLUT1, GAD65, Gephyrin) and dendritic injury (change in the spine- numbers and sub-types). Overall, these microglial EVs carrying NLRP3 cargoes can cause synaptodendritic injury resulting in HAND in patients via microglia-neuron cross talk. NLRP3: NOD-, LRR- and pyrin domain-containing protein 3; ASC: apoptosis-associated speck-like protein; IL 1β: interleukin 1β; PSD95: postsynaptic density protein 95; vGLUT1: vesicular glutamate transporters; GAD65: glutamic acid decarboxylase; Tat: trans-activator of transcription.

To study the functional alterations in neurons induced by the HPM-exosomes, we recorded miniature excitatory postsynaptic currents (mEPSCs) in rat primary hippocampal neurons (DIV 18-22) treated with scrambled siRNA-MDEV, scrambled siRNA+-Tat-MDEV, NLRP3 siRNA MDEV, NLRP3 siRNA+Tat MDEVs isolated from HPM. As demonstrated in Figure 6A-C, scrambled siRNA+-Tat-MDEVs significantly (*P* < 0.05) decreased the excitatory neurotransmission (reduced frequency and amplitude) in rat primary neurons. On the other hand, in rat primary neurons exposed to NLRP3 siRNA MDEVs, NLRP3 siRNA+Tat MDEVs, the mEPSCs were comparable to the neurons exposed to scrambled siRNA-MDEV; however, the amplitudes still remained low [Figure 6C].

### Schematic representation of microglia-neuronal cross-talk in synaptodendritic injury involving Tat MDEVs

**Figure 7:**
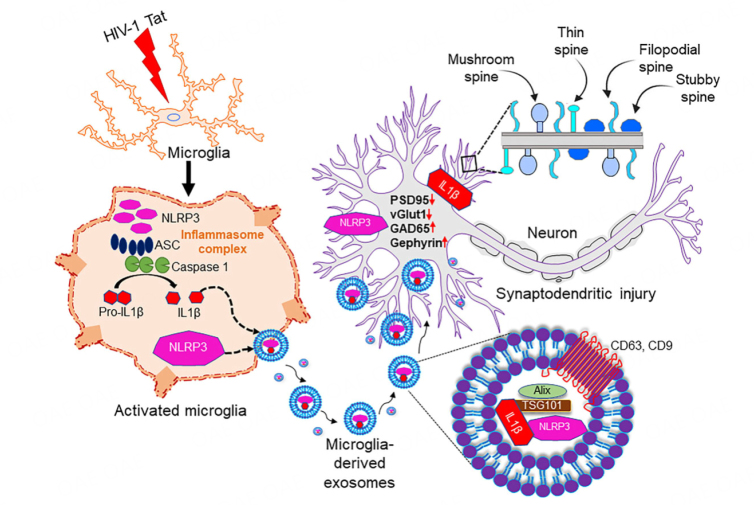
Schematic representation of the role of microglial NLRP3 on neuronal synaptodendritic injury via exosomes. Exposure of microglia (BV2 or HPM) with HIV-1 Tat results in the activation of NLRP3 inflammasome complex, whichleads to the production of IL-1β and microglial activation. Thereafter, these NLRP3 and IL1β can be packaged in the exosomes and released by the microglia. These exosomes carrying NLRP3/IL1β upon uptake by the neurons result in alteration of synaptic proteins (PSD95, vGLUT1, GAD65, Gephyrin) and dendritic injury (change in the spine- numbers and sub-types). Overall, these microglial EVs carrying NLRP3 cargoes can cause synaptodendritic injury resulting in HAND in patients via microglia-neuron cross talk. NLRP3: NOD-, LRR- and pyrin domain-containing protein 3; ASC: apoptosis-associated speck-like protein; IL 1β: interleukin 1β; PSD95: postsynaptic density protein 95; vGLUT1: vesicular glutamate transporters; GAD65: glutamic acid decarboxylase; Tat: trans-activator of transcription.

In this study, we demonstrated that exposure of microglia (BV2/HPM) to HIV-1 Tat resulted in the release of MDEVs carrying NLRP3 and IL1β cargoes, which could be taken up by the neurons. Upon uptake by the neurons of the NLRP3, IL1β containing MDEV cargoes, there was a decreased expression of synaptic proteins PSD95, excitatory vGLUT1, and an increase in inhibitory synaptic proteins - GAD65 and Gephyrin. Further, these MDEVs also resulted in a loss of dendric spines as well as a change in the ratio of spine sub-types (mushroom, stubby, filopodia, thin). Silencing of microglial NLRP3 led to the protection of Tat MDEV mediated neuronal synaptodendritic injury [Figure 7]. Overall, the Tat-MDEVs carrying NLRP3 cargoes could mediate neuronal synaptodendritic injury underlying HAND involving the microglial-neuronal cross-talk [Figure 7].

## DISCUSSION

HAND is a common cause of morbidity in HIV-1 positive individuals who are on cART^[42]^. The prevalence of milder forms of the disease, such as asymptomatic neurocognitive impairment (ANI) or mild-neurocognitive disorder (MND), however, continues to increase, accounting for ~70% of HAND cases^[42]^. As demonstrated by several investigators, people living with HIV-1 on cART continue to exhibit neuronal damage^[43]^. Although neurons are less susceptible to direct infection, infected microglia can mediate neuronal damage involving both the EVs and non-EV fractions^[44-47]^. In healthy cells, microglia secrete EVs to support the metabolic functions of neurons and to provide them with substrates needed for energy metabolism during synaptic activity^[21-23]^. Ample evidence suggests that EVs play a significant role in microglia-mediated neuroinflammation and the progression of several neurodegenerative disorders in the brain^[27,48,49]^. Recent studies indicate that EVs are key players in intercellular communication that underlies physiological processes such as synaptic plasticity and maintenance of myelination^[50,51]^. Similarly, MDEVs also affect neuronal functions by delivering proinflammatory cytokines and other neurotoxic mediators under pathological conditions^[26,27]^. 

As reviewed by Saylor *et al*. (2016), evidence suggests that inflammation plays a critical role in HAND^[42]^. HIV-1 Tat protein has been shown to be present in the brains of infected individuals and is an important contributor to the development of HAND^[52-55]^. In our previous study, we showed that HIV-1 Tat-mediated microglial activation involves the NLRP3 inflammasome pathway. In brief, our previous findings showed that Tat primes and activates the NLRP3 inflammasome in microglia, resulting in the release of IL-1β, a highly potent cytokine that, in turn, induces other cytokines, including IL-6 and TNF-α, to further exacerbate neuroinflammation^[36]^. Other investigators have also shown induction of the NLRP3 inflammasome in both microglia and monocytes during HIV-1 infection^[56-58]^. Interestingly, individuals who developed ANI and MND have elevated levels of NLRP3 activators such as ceramide and multiple forms of cholesterol compared with cognitively normal HIV-1-positive individuals^[59]^. In the present study, we demonstrate that Tat-induced NLRP3 in the microglia can be packaged in MDEVs and is released in the extracellular space. The MDEVs carrying the NLRP3 cargoes can be taken up by the neurons, in turn leading to synaptodendritic injury and functional impairment [Figure 7]. Although NLRP3 is primarily induced by microglia, recent reports have also demonstrated the role of neuronal NLRP3 in Parkinson’s Disease^[41]^. There is, however, no evidence of activation of NLRP3 in neurons in HIV-1 to date. While the role of microglial NLRP3 in neuronal damage has been demonstrated in the presence of HIV protein gp120^[60]^, the role of Tat in microglial NLRP3 mediated neuronal damage remains elusive.

In the present study, we demonstrated that Tat exposed MDEVs had a size distribution ranging from 50-150 nm and were found to carry the NLRP3 and IL1β cargoes. The numbers of MDEVs, however, did not change in the presence or absence of Tat. The current study was not aimed at assessing the direct role of IL1β on neuronal injury, instead was focused on the indirect effect of activation of the microglial NLRP3 pathway. Exposure of neurons to Tat-MDEVs resulted in downregulation of synaptic proteins- PSD95, synaptophysin, excitatory vGLUT1 and upregulation of inhibitory proteins- Gephyrin, GAD65, thus suggesting impaired neuronal transmissibility. Furthermore, Tat MDEVs exposed neurons also exhibited decreased mEPSCs, thereby implicating functional impairment of the neurons. Previous studies have demonstrated damage of the pyramidal neurons in the neocortex during HIV infection with alterations in excitatory neurotransmitters and inflammatory markers^[43]^. Interestingly, it was also shown that PSD95 expression was downregulated^[61]^, and Gephyrin expression was increased in neurons following Tat exposure^[17]^. Other reports in HIV transgenic mice have demonstrated an increase in Gephryin, associated with inhibitory transmission and minimal dendritic pathology^[62]^. Dysregulation of excitatory/inhibitory proteins^[62,63] ^could underlie functional impairment of the neurons, as evidenced by the increase in mEPSCs in our study. Previous reports from our laboratory have also shown that HIV-1 Tat could also induce alteration of EV cargoes from astrocytes, in turn leading to impairment of the synaptic architecture of neurons^[28,29]^.

Alterations of synaptic proteins and cognitive deficits are often associated with a neuronal spine injury, as shown in HIV-Tg rats^[64]^. Our present study showed loss of dendritic spines, mature spine sub-types mushroom and stubby, following exposure of neurons with Tat-MDEVs. Alterations in total spine density have been demonstrated by several investigators in HAND^[62-64]^. In line with our study, clinical studies have shown that HIV patients exhibit loss of neurons, and aberrant sprouting, and dystrophic synaptodendritic connections in the CNS^[65]^, with decreased expression of MAP2 and neurofilament, and markers for synaptodendritic connectivity. Intriguingly, it has also been reported that damage initiates in the synapses and dendrites and then spreads to the rest of the neuron, leading to apoptosis^[66,67]^. Association of alterations of spine sub-types with synaptodendritic injury has not been reported earlier in HAND. Additionally, the role of MDEVs in the process of synaptodendritic injury is a novel finding of this study. Next, to assess the role of microglial NLRP3 in this process, neurons were exposed to MDEVs from NLRP3 silenced microglia in the presence of Tat. Results showed that these MDEVs, derived from NLRP3 silenced microglia, abrogated Tat-MDEV mediated neurotoxicity as evidenced by restoration of changes in synaptic proteins- PSD95, Synaptophysin, GAD65, and Gephyrin as well as total spines and spine sub-types. The protective role was also observed in the frequency of the mEPSCs, but not in the amplitudes. 

To summarize, this study demonstrated that HIV-1 Tat exposure can lead to the release of MDEVs from microglia, carrying NLRP3 cargoes. These MDEVs, upon being taken up by the neurons, resulted in synaptodendritic injury and functional impairment- as evidenced by decreased mEPSCs. The role of microglia-neuronal cross-talk via MDEVs has not been demonstrated earlier in the context of HAND; specifically, how the microglial NLRP3 plays a role in this process will open future avenues for the development of adjunctive therapeutics for HAND. Although we demonstrated the role of microglial NLRP3 in neuronal injury, further studies are warranted to assess the mechanistic underpinnings by which MDEVs mediate neuronal damage. The role of NLRP3 in inflammation is very well known; however, the role of the same NLRP3 in neuronal damage is an interesting finding that implicates the role of the therapeutic potential of NLRP3 blockers as a treatment option for HAND and other neuroinflammatory conditions.

## DECLARATIONS

### Availability of data and materials

Data and materials will be available on request.

### Copyright

© The Author(s) 2022.

## References

[1] Joseph SB, Arrildt KT, Sturdevant CB, Swanstrom R (2015). HIV-1 target cells in the CNS. J Neurovirol.

[2] Kranick SM, Nath A (2012). Neurologic complications of HIV-1 infection and its treatment in the era of antiretroviral therapy. Continuum (Minneap Minn).

[3] Rojas-Celis V, Valiente-Echeverría F, Soto-Rifo R, Toro-Ascuy D (2019). New challenges of HIV-1 infection: how HIV-1 attacks and resides in the central nervous system. Cells.

[4] Kusdra L, McGuire D, Pulliam L (2002). Changes in monocyte/macrophage neurotoxicity in the era of HAART: implications for HIV-associated dementia. AIDS.

[5] Debaisieux S, Rayne F, Yezid H, Beaumelle B (2012). The ins and outs of HIV-1 Tat. Traffic.

[6] Schwarze SR, Ho A, Vocero-Akbani A, Dowdy SF (1999). In vivo protein transduction: delivery of a biologically active protein into the mouse. Science.

[7] Roy S, Delling U, Chen CH, Rosen CA, Sonenberg N (1990). A bulge structure in HIV-1 TAR RNA is required for Tat binding and Tat-mediated trans-activation. Genes Dev.

[8] Rice AP (2017). The HIV-1 Tat protein: mechanism of action and target for HIV-1 cure strategies. Curr Pharm Des.

[9] Garden GA (2002). Microglia in human immunodeficiency virus-associated neurodegeneration. Glia.

[10] Churchill MJ, Wesselingh SL, Cowley D (2009). Extensive astrocyte infection is prominent in human immunodeficiency virus-associated dementia. Ann Neurol.

[11] Brack-Werner R (1999). Astrocytes: HIV cellular reservoirs and important participants in neuropathogenesis. AIDS.

[12] Sabatier JM, Vives E, Mabrouk K (1991). Evidence for neurotoxic activity of tat from human immunodeficiency virus type 1. J Virol.

[13] Conant K, Tornatore C, Atwood W, Meyers K, Traub R, Major E (1994). In vivo and in vitro infection of the astrocyte by HIV-1. Advances in Neuroimmunology.

[14] Tornatore C, Meyers K, Atwood W, Conant K, Major E (1994). Temporal patterns of human immunodeficiency virus type 1 transcripts in human fetal astrocytes. J Virol.

[15] Liu Y, Jones M, Hingtgen CM (2000). Uptake of HIV-1 tat protein mediated by low-density lipoprotein receptor-related protein disrupts the neuronal metabolic balance of the receptor ligands. Nat Med.

[16] King JE, Eugenin EA, Buckner CM, Berman JW (2006). HIV tat and neurotoxicity. Microbes Infect.

[17] Hargus NJ, Thayer SA (2013). Human immunodeficiency virus-1 Tat protein increases the number of inhibitory synapses between hippocampal neurons in culture. J Neurosci.

[18] Santerre M, Bagashev A, Gorecki L (2019). HIV-1 Tat protein promotes neuronal dysregulation by inhibiting E2F transcription factor 3 (E2F3). J Biol Chem.

[19] Marinelli S, Basilico B, Marrone MC, Ragozzino D (2019). Microglia-neuron crosstalk: signaling mechanism and control of synaptic transmission. Semin Cell Dev Biol.

[20] Matejuk A, Ransohoff RM (2020). Crosstalk between astrocytes and microglia: an overview. Front Immunol.

[21] Potolicchio I, Carven GJ, Xu X (2005). Proteomic analysis of microglia-derived exosomes: metabolic role of the aminopeptidase CD13 in neuropeptide catabolism. J Immunol.

[22] Fauré J, Lachenal G, Court M (2006). Exosomes are released by cultured cortical neurones. Mol Cell Neurosci.

[23] Upadhya R, Zingg W, Shetty S, Shetty AK (2020). Astrocyte-derived extracellular vesicles: neuroreparative properties and role in the pathogenesis of neurodegenerative disorders. J Control Release.

[24] Delpech JC, Herron S, Botros MB, Ikezu T (2019). Neuroimmune crosstalk through extracellular vesicles in health and disease. Trends Neurosci.

[25] You Y, Ikezu T (2019). Emerging roles of extracellular vesicles in neurodegenerative disorders. Neurobiol Dis.

[26] Yang Y, Boza-Serrano A, Dunning CJR, Clausen BH, Lambertsen KL, Deierborg T (2018). Inflammation leads to distinct populations of extracellular vesicles from microglia. J Neuroinflammation.

[27] Aires ID, Ribeiro-Rodrigues T, Boia R (2021). Microglial extracellular vesicles as vehicles for neurodegeneration spreading. Biomolecules.

[28] Dagur RS, Liao K, Sil S (2020). Neuronal-derived extracellular vesicles are enriched in the brain and serum of HIV-1 transgenic rats. J Extracell Vesicles.

[29] Sil S, Singh S, Chemparathy DT, Chivero ET, Gordon L, Buch S (2021). Astrocytes & astrocyte derived extracellular vesicles in morphine induced amyloidopathy: implications for cognitive deficits in opiate abusers. Aging Dis.

[30] Apcher G, Heink S, Zantopf D (2003). Human immunodeficiency virus-1 Tat protein interacts with distinct proteasomal α and β subunits. FEBS Letters.

[31] Sil S, Niu F, Tom E, Liao K, Periyasamy P, Buch S (2019). Cocaine mediated neuroinflammation: role of dysregulated autophagy in pericytes. Mol Neurobiol.

[32] McCarthy MK, Weinberg JB (2015). The immunoproteasome and viral infection: a complex regulator of inflammation. Front Microbiol.

[33] Thangaraj A, Periyasamy P, Liao K (2018). HIV-1 TAT-mediated microglial activation: role of mitochondrial dysfunction and defective mitophagy. Autophagy.

[34] Minakaki G, Menges S, Kittel A (2018). Autophagy inhibition promotes SNCA/alpha-synuclein release and transfer via extracellular vesicles with a hybrid autophagosome-exosome-like phenotype. Autophagy.

[35] Leidal AM, Debnath J (2021). Emerging roles for the autophagy machinery in extracellular vesicle biogenesis and secretion. FASEB Bioadv.

[36] Chivero ET, Guo ML, Periyasamy P, Liao K, Callen SE, Buch S (2017). HIV-1 Tat primes and activates microglial NLRP3 inflammasome-mediated neuroinflammation. J Neurosci.

[37] Arikkath J, Peng IF, Ng YG (2009). Delta-catenin regulates spine and synapse morphogenesis and function in hippocampal neurons during development. J Neurosci.

[38] Beaudoin GM 3rd, Lee SH, Singh D (2012). Culturing pyramidal neurons from the early postnatal mouse hippocampus and cortex. Nat Protoc.

[39] Hu G, Niu F, Liao K (2020). HIV-1 Tat-induced astrocytic extracellular vesicle miR-7 impairs synaptic architecture. J Neuroimmune Pharmacol.

[40] Song L, Pei L, Yao S, Wu Y, Shang Y (2017). NLRP3 inflammasome in neurological diseases, from functions to therapies. Front Cell Neurosci.

[41] von Herrmann KM, Salas LA, Martinez EM (2018). NLRP3 expression in mesencephalic neurons and characterization of a rare NLRP3 polymorphism associated with decreased risk of Parkinson’s disease. NPJ Parkinsons Dis.

[42] Saylor D, Dickens AM, Sacktor N (2016). HIV-associated neurocognitive disorder--pathogenesis and prospects for treatment. Nat Rev Neurol.

[43] Kovalevich J, Langford D (2012). Neuronal toxicity in HIV CNS disease. Future Virol.

[44] Alvarez-Carbonell D, Ye F, Ramanath N (2019). Cross-talk between microglia and neurons regulates HIV latency. PLoS Pathog.

[45] Borrajo A, Spuch C, Penedo MA, Olivares JM, Agís-Balboa RC (2021). Important role of microglia in HIV-1 associated neurocognitive disorders and the molecular pathways implicated in its pathogenesis. Ann Med.

[46] Ru W, Tang SJ (2017). HIV-associated synaptic degeneration. Mol Brain.

[47] Ru W, Liu X, Bae C (2019). Microglia mediate HIV-1 gp120-induced synaptic degeneration in spinal pain neural circuits. J Neurosci.

[48] Guo M, Hao Y, Feng Y (2021). Microglial exosomes in neurodegenerative disease. Front Mol Neurosci.

[49] Paolicelli RC, Bergamini G, Rajendran L (2019). Cell-to-cell communication by extracellular vesicles: focus on microglia. Neuroscience.

[50] Holm MM, Kaiser J, Schwab ME (2018). Extracellular vesicles: multimodal envoys in neural maintenance and repair. Trends Neurosci.

[51] Schnatz A, Müller C, Brahmer A, Krämer-Albers EM (2021). Extracellular vesicles in neural cell interaction and CNS homeostasis. FASEB Bioadv.

[52] (2000). Valle L, Croul S, Morgello S, Amini S, Rappaport J, Khalili K. Detection of HIV-1 Tat and JCV capsid protein, VP1, in AIDS brain with progressive multifocal leukoencephalopathy. J Neurovirol.

[53] Hudson L, Liu J, Nath A (2000). Detection of the human immunodeficiency virus regulatory protein tat in CNS tissues. J Neurovirol.

[54] Li W, Li G, Steiner J, Nath A (2009). Role of Tat protein in HIV neuropathogenesis. Neurotox Res.

[55] Johnson TP, Patel K, Johnson KR (2013). Induction of IL-17 and nonclassical T-cell activation by HIV-Tat protein. Proc Natl Acad Sci U S A.

[56] Guo H, Gao J, Taxman DJ, Ting JP, Su L (2014). HIV-1 infection induces interleukin-1β production via TLR8 protein-dependent and NLRP3 inflammasome mechanisms in human monocytes. J Biol Chem.

[57] Hernandez JC, Latz E, Urcuqui-Inchima S (2014). HIV-1 induces the first signal to activate the NLRP3 inflammasome in monocyte-derived macrophages. Intervirology.

[58] Walsh JG, Reinke SN, Mamik MK (2014). Rapid inflammasome activation in microglia contributes to brain disease in HIV/AIDS. Retrovirology.

[59] Bandaru VV, Mielke MM, Sacktor N (2013). A lipid storage-like disorder contributes to cognitive decline in HIV-infected subjects. Neurology.

[60] He X, Yang W, Zeng Z (2020). NLRP3-dependent pyroptosis is required for HIV-1 gp120-induced neuropathology. Cell Mol Immunol.

[61] Kim HJ, Martemyanov KA, Thayer SA (2008). Human immunodeficiency virus protein Tat induces synapse loss via a reversible process that is distinct from cell death. J Neurosci.

[62] Fitting S, Ignatowska-Jankowska BM, Bull C (2013). Synaptic dysfunction in the hippocampus accompanies learning and memory deficits in human immunodeficiency virus type-1 Tat transgenic mice. Biol Psychiatry.

[63] Marks WD, Paris JJ, Schier CJ (2016). HIV-1 Tat causes cognitive deficits and selective loss of parvalbumin, somatostatin, and neuronal nitric oxide synthase expressing hippocampal CA1 interneuron subpopulations. J Neurovirol.

[64] Roscoe RF Jr, Mactutus CF, Booze RM (2014). HIV-1 transgenic female rat: synaptodendritic alterations of medium spiny neurons in the nucleus accumbens. J Neuroimmune Pharmacol.

[65] Ellis R, Langford D, Masliah E (2007). HIV and antiretroviral therapy in the brain: neuronal injury and repair. Nat Rev Neurosci.

[66] Everall IP, Heaton RK, Marcotte TD (1999). Cortical synaptic density is reduced in mild to moderate human immunodeficiency virus neurocognitive disorder. HNRC Group. HIV Neurobehavioral Research Center. Brain Pathol.

[67] Masliah E, Heaton RK, Marcotte TD (1997). Dendritic injury is a pathological substrate for human immunodeficiency virus-related cognitive disorders. HNRC Group. The HIV Neurobehavioral Research Center. Ann Neurol.

